# Supplementary material for: Transcriptomic Analysis of the Molecular Mechanism Potential of Grafting—Enhancing the Ability of Oriental Melon to Tolerate Low-Nitrogen Stress
Source: Int J Mol Sci. 2024 Jul 27;25(15):8227. doi: 10.3390/ijms25158227 (PMC11311868; doi:10.3390/ijms25158227)
Supplement: Supplementary file 1 [file ijms-25-08227-s001.zip › ijms-3051449-supplementary.pdf]

Table S1 Primers for the qRT-PCR assays.

**Gene name**	**Primer sequence**
MELO3C008287.2	F: GAACGCTTCGTGTGCAGTTC
	R: CCACCGCAGAGTTAATGGGT
MELO3C002144.2	F: CCAGAGTTGCTGGTTACCGT
	R: GTGTAGGGGCCTTTCCTGTC
MELO3C010836.2	F: CGGAATCGACAAGGGTAATGTG
	R: ATGAAACCGACGAAGCCACT
MELO3C018476.2	F: TTGCGAGCGTGTTCTTCTCT
	R: TCGTTGGAGCTTTCACTGCT
MELO3C004373.2	F: TGCCGATTCGGTTGAGGAAA
	R: TTTCTCCGTCTCCAAGCGAC
MELO3C022373.2	F: TCGATTTCTTGCACCGACGA
	R: AAAGCCCGGACGTTGATCTT
MELO3C014430.2	F: CGTTCCAACCTCCGTCTCAA
	R: GGCTCCTCTGTGGTGAACTC
MELO3C007574.2	F: ATGCCATTCACCCGTTCCAT
	R: TGCCTGCTTCGACGTAGTTT
MELO3C014002.2	F: ATGCGTTTTCGGGTTTGGTG
	R: TGAAATGGTGCAGCAACGTG
MELO3C007734.2	F: GGCTCCCAAAATGGGATGGA
	R: AGCAAAAGCAGGAGGTGAGG
